# Supplementary material for: After Ischemic Stroke, Minocycline Promotes a Protective Response in Neurons via the RNA-Binding Protein HuR, with a Positive Impact on Motor Performance
Source: Int J Mol Sci. 2023 May 29;24(11):9446. doi: 10.3390/ijms24119446 (PMC10253390; doi:10.3390/ijms24119446)
Supplement: Supplementary file 1 [file ijms-24-09446-s001.zip › ijms-2401708-supplementary.pdf]

Supplemental materials:

.

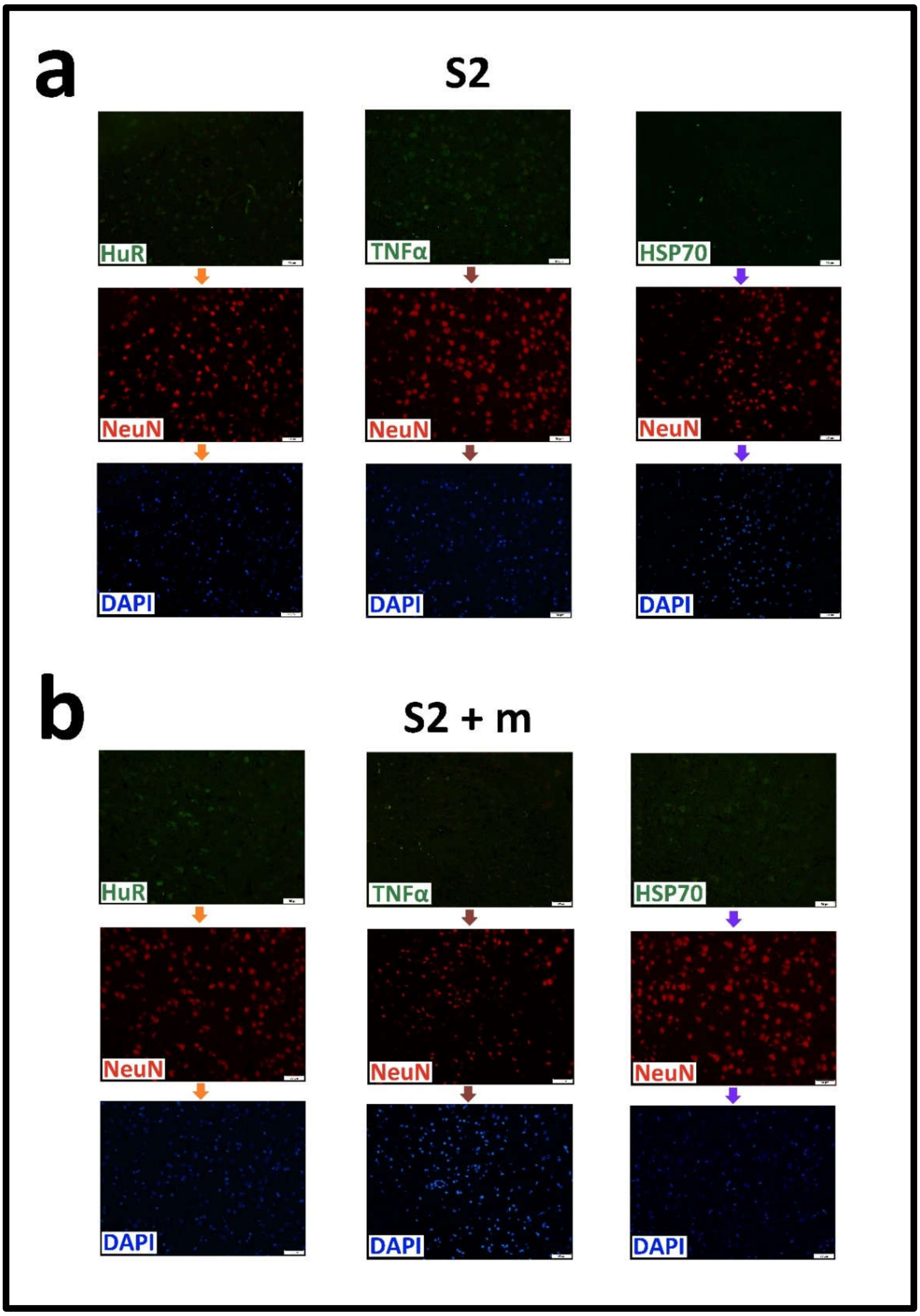

Figure S1. Effect of minocycline on selected parameters of inflammation after induction of ischemic stroke in rats. Immunohistochemical staining. **(a)** Representative images for S2 group. The double-immunolabeled (NeuN-HuR; NeuN-HSP70; NeuN-TNFα) coronal section at the infarct boundary in Ipsi2 region. The infarct is visible in the lower part of the image; Scale bar = 50 µm **(b)** Representative images for S2+m group. The double-immunolabeled (NeuN-HuR; NeuN-TNFα; NeuN-HSP70;) coronal section at the infarct boundary in Ipsi2 re-gion. The infarct is visible in the lower part of the image; Scale bar = 50 µm.
